# OpenAlex

**DOI:** 10.29173/jchla29885

**Published:** 2025-12-01

**Authors:** Jack Young, Zahra Premji

**Affiliations:** 1 McMaster University Libraries Hamilton, ON, Canada; 2 University of Victoria Libraries Victoria, BC, Canada

**Product:** OpenAlex

**URL:** https://openalex.org/

## Purpose

The purpose of this review is to describe the utility of the open bibliographic database OpenAlex. In addition to providing a collaborative overview of the product, the individual authors of this review will explore OpenAlex’s value as a source of open linked bibliometric data (JY) and its use for systematic literature searching and evidence synthesis (ZP). Due to space limits, it was not possible to provide a complete assessment of all features, strengths, and weaknesses of OpenAlex.

## Product/resource description

OpenAlex describes itself as “a catalog of works”, specifically of scholarly outputs which extend beyond the research article to include datasets, books, dissertations, preprints, and other formats [1]. In addition to works, OpenAlex also generates records for three other distinct entity types: Authors, Sources, and Institutions. Beyond cataloguing these works, OpenAlex also tracks connections between the works. Unlike most subscription products, OpenAlex’s dataset is open and available for download and reuse under a Creative Commons Zero (CC0) license.

## Intended audience/users

As one of the largest openly accessible databases of scholarly works with a web application, OpenAlex is suitable for all scholarly researchers, but especially those in resource-constrained environments who may not have access to paid subscription databases. OpenAlex’s linked data structure and open application programming interface (API) also make it suitable for audiences engaged in bibliometric analysis and research impact assessment activities.

## Platform

**Fig. 1:**
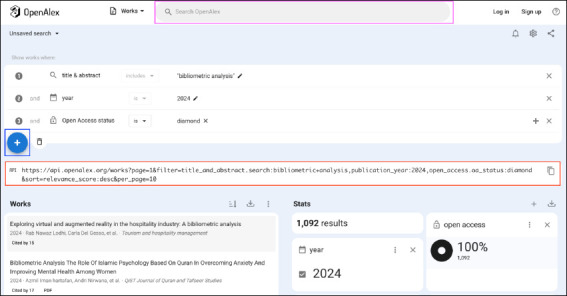
OpenAlex’s main search / results page. Highlights: main search box (pink box at the top of the figure); + button to add search filters (blue box in the middle of the figure on the left side); auto-generated API call (red box in the middle of the figure).

The web application (Figure 1) uses a single search box design, with an auto-complete feature. Users can type in their keywords to conduct a search, or type the name of a metadata field or filter, as OpenAlex refers to them (e.g. Author, Source, Institution), to begin a search query using that filter. Alternatively, hitting the Enter button without typing in any terms will send the user to the results screen with a blank search. From there, there are multiple ways to add lines to a query, including a search box at the top of the page, or a + symbol further down on the page from which filters can be added. This way of searching may feel unintuitive to users familiar with subscription databases that use drop-down search field options. However, after one uses the web application a couple of times, it quickly becomes familiar. On the user interface (UI) on the web, OpenAlex offers 51 filters.

OpenAlex also offers a representational state transfer (REST) API that is free and requires no authentication (up to 100 000 requests per user per day), making it accessible for a wide variety of use cases. Detailed documentation [2] and a small collection of pre-built Jupytr Notebook tutorials [3] make it easy to get started on a variety of common research impact tasks (including citation tracking, collaboration analysis, and open access monitoring). OpenAlex also makes it easy to move queries from the UI to the API, as all searches performed within the UI are automatically translated into an API query that can be made visible within the UI’s settings menu (Figure 1). Content in the free API is updated monthly via a data dump.

## Currency/coverage

Building off the complete dataset of the now defunct Microsoft Academic Graph, OpenAlex aggregates content daily from multiple open data sources across the web. These include article databases (e.g. PubMed), institutional and subject-specific repositories (e.g. PubMed Central, arXiv, Zenodo), and specialized metadata sources (e.g. Open Contributor & Researcher ID (ORCiD) Directory of Open Access Journals (DOAJ), Research Organization Registry (RoR)). Chief among these sources is CrossRef, the Digital Object Identifier (DOI) registry responsible for assigning persistent identifiers to works published in the vast majority of scholarly journals. These diverse data sources pull a wide variety of work types into OpenAlex, including journal articles, conference proceedings, datasets, preprints, and even LibGuides.

While this makes OpenAlex one of the largest and most up to date databases currently available (indexing “over 240m works, with tens of thousands added daily” [4]), it is important to also acknowledge the potential drawbacks of this approach. Without an internal content evaluation process, OpenAlex relies heavily on the quality of its data sources. While a data source like PubMed has clear and standardized journal selection processes, far less quality control is applied to content coming from sources like CrossRef and Zenodo, making OpenAlex result sets vulnerable to potentially questionable content and messy metadata.

### Bibliometric data

The bibliometric data contained in OpenAlex makes it a potentially valuable tool for research impact analysis and projects utilizing bibliometric methodologies.

### Search functions

All four of OpenAlex’s distinct entity types can be used for bibliometric analysis. “Sources” and “Institutions” records may be of some value if a user is interested in a snapshot of the overall output of a specific journal or research organization. The “Authors” entity, which attempts to create algorithmically-generated researcher profiles for each distinct author represented in the database, could have significant utility if its accuracy were improved, as discussed below. Currently, the “Works” entity is the most valuable for bibliometric analysis, as it contains metadata for each unique research output within the database. Users can couple free text search and OpenAlex’s comprehensive search filters to gather a highly specific result set for bibliometric analysis. Common filters like author, publication year, and open access status are supplemented by more specialized filters like citation percentile, estimated article processing charge (APC) paid, and corresponding author institution that make OpenAlex unique amongst other bibliometric databases. One missing feature that would enhance OpenAlex’s value as a bibliometric tool is the ability to search for a list of DOIs. This would allow users to quickly and confidently recreate result sets generated in external databases for deeper analysis within OpenAlex.

### Data export

Although the user interface offers a “Stats” section that provides some basic bibliometric indicators for result sets (e.g. total citations, open access %), noticeably absent are useful metrics like citation rates year-over-year and field normalized citation rates. The addition of such features to the user interface could make OpenAlex more appealing to users interested in leveraging its bibliometric insights without having to work with the raw data itself.

**Fig. 2:**
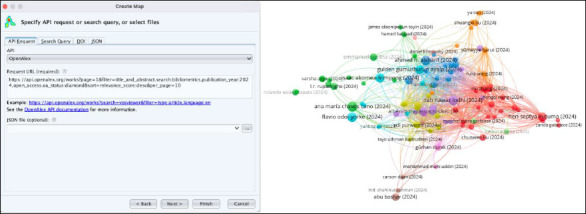
OpenAlex API integration within VOSviewer network visualization software

Currently, comprehensive bibliometric analysis requires the export of OpenAlex data to external tools. The OpenAlex user interface enables this by allowing direct export of up to 100 000 records to a CSV file, a number that far exceeds limits set by its subscription-based counterparts. The format of this data, once exported, makes it relatively easy to use within external analysis tools (e.g. Excel, R). Further, an API integration with the bibliometric visualization software, VOSviewer, makes it easy to create compelling network visualizations of OpenAlex data (Figure 2).

### Data quality

Current perspectives on responsible research assessment stress the importance of considering a wide variety of research outputs when evaluating research impact [5-7]. Research impact assessments performed in OpenAlex can pull together publications, datasets, pre-prints, conference material, and more, allowing for a more complete understanding of impact than that offered by databases solely indexing journal-based content. It should be noted that patents are not currently indexed in OpenAlex. OpenAlex’s primary comparator in this space, The Lens, is currently a better option for users interested in exploring the impact of research on intellectual property.

OpenAlex’s broad approach to collection and indexing is also reflected in the metadata fields available for each record. Of particular note is the estimated APC field. Leveraging data from DOAJ, this field enables analysis of APC costs paid by researchers at an institution–a particularly valuable feature given the growth of transformative (read-publish) agreements that offer discounted or waived APC costs for an institution’s researchers.

**Fig. 3:**
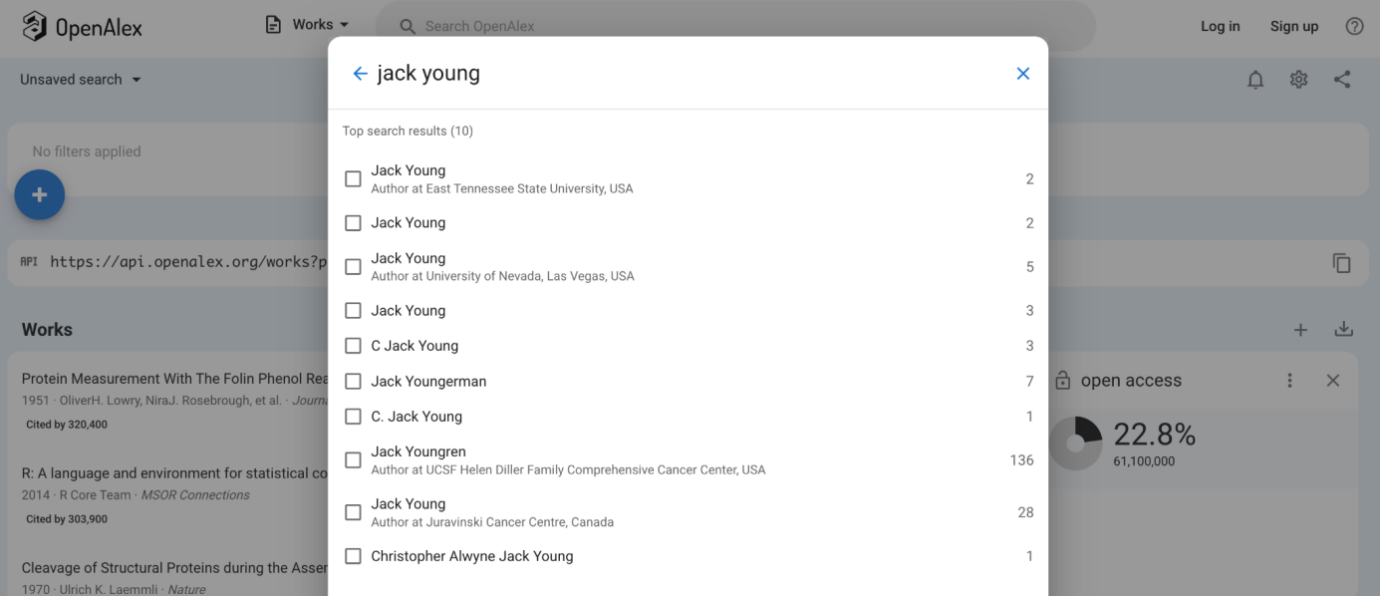
If an author’s profile does not appear in the top 10 results, it cannot be used to filter result sets in the UI

This broad scope, however, does have drawbacks. The algorithm-driven name disambiguation (the process of distinguishing unique authors from one another within the database) struggles significantly with common names, frequently causing a single author’s works to be spread out across multiple profiles or multiple authors’ works to be subsumed under one profile. This issue is exacerbated by the fact that only the top 10 author profiles are displayed when filtering works results by author, meaning that those with exceedingly common names may not be able to find their work at all (Figure 3).

Issues with the consistency of some metadata fields can also hamper certain analyses. For example, the “Corresponding Institution” (a field with great utility for institutions assessing the cost of open access fees) is currently missing from a significant portion of OpenAlex’s records. As of this writing, OpenAlex is aware of this issue and is working toward its resolution.

#### Evidence synthesis

Note that the following section on evidence synthesis is about the user interface only. It is possible that certain functions may be available in the API interface but not currently available in the user interface.

### Search functions

Search systems should have a number of functions in order to be suitable for comprehensive searching conducted for evidence synthesis. Some of the necessary or essential features, according to either Gusenbauer & Haddaway [8] or Bethel & Rogers [9], are shown in Table 1 below.

**Table 1:** A list of selected (not all) essential or necessary features from Bethel & Rogers [9] and Gusenbauer & Haddaway’s [8] lists, and the presence or absence of these features in OpenAlex. Note: this is not intended to be a full technical assessment of OpenAlex.

Feature	Essential feature in Bethel & Rogers [9]	Necessary feature in Gusenbauer & Haddaway [8]	Found in OpenAlex (web interface)
Searching by field	✔	✔	✔
Boolean (OR, AND, and NOT)	✔	✔	✔
Phrase searching	✔	✔	✔
Nesting parenthesis	✔	✔	✔
Maximum/high number of accessible hits		✔	✔
Right-hand truncation	✔		✖
Proximity/adjacency terms	✔		✖
Command line searches	✔		✖
Line-by-line searching	✔		✖
Combining strings	✔		✖
Viewable search history	✔		✖
Ability to handle long searches	✔	✔	✔
Controlled vocabulary	✔		✖
Reproducibility/consistency of search (time and location)	✔	✔	✔

OpenAlex has a number of these important features (Table 1) as well as a high export limit. Among the missing features related to search query construction, the most notable is the lack of truncation and proximity searching. The lack of truncation will result in longer search strings, especially for variations that wouldn’t naturally be included by OpenAlex’s automatic stemming (for example, drug names which sometimes have (R) or TM appended to the end of the name). According to OpenAlex’s documentation, the search query has a 2048 character limit for the entire URL-based query [10]. Despite the stated character limit, a test search query of 2800 characters (containing both AND and ORs and with three levels of nested parentheses) was successfully executed by OpenAlex in May 2025. There is an upper limit, as larger (5000 characters) test searches did not run successfully, though the exact limit is unclear. Evidence synthesis searches can easily exceed this query length, especially for complex topics that require either a greater number of keywords or multiple variants for each term due to the missing truncation operator.

**Fig. 4:**
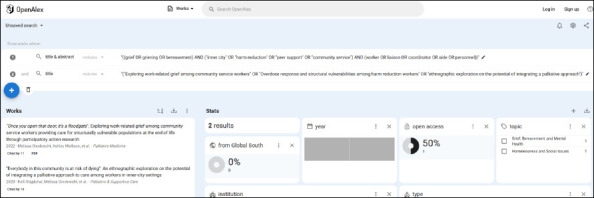
A two-line search with the search string in line 1 (searched using the “Title & Abstract” filter) and the seed article titles in line 2 to verify retrieval of seed articles.

It is possible to create multiple search lines in OpenAlex, each using the “Title” or “Title & Abstract” filters. However, each subsequent search line can only be added from the search box at the top of the results page (see pink box at the top of Figure 1). Even though there is a + symbol below the first search line in the results menu that lets you add additional lines with filters (outlined by a blue box in the centre left of Figure 1), the “Title” or “Title & Abstract” filters will be greyed out if they have already been used in the search thus preventing their re-use. This appears to be the case for the “Title” and “Title & Abstract” filters, but not other filters such as institution or type. Each subsequent line gets added to the existing ones using an AND, and this cannot be changed. So, while it is possible to create a two-concept search over two search lines–as long as they need to be combined with an AND–it is not possible to combine select lines (and skip others) or combine two lines with an OR, as is possible in other subscription database platforms (such as Web of Science or Ovid). If various combinations of AND and OR are required between search terms, it can only be done using nested parentheses and Boolean operators within a single line and thus within a single filter. However, the ability to combine multiple lines with the AND operator makes it possible to test the retrieval of seed or known articles against a search (Figure 4). The number of results shown on the screen is always for the combined search lines (ANDed together by default), and the number of results for each line separately is not available. Searchers who want to know the number of results of two different search query iterations will need to note this information after the first test, and delete the first query before testing the second, otherwise the lines will be ANDed together.

A final feature of interest to evidence searchers is the ability to create a free account, create alerts, and save searches for re-running at a later date.

A search syntax sheet that includes OpenAlex among its listed sources is available, co-created by one of the authors of this review [11]. These types of syntax sheets are created through testing and with reference to database documentation, and provide details of search features of each search platform and how similar functions translate across platforms.

### Data content, exporting, and quality

In addition to the CSV format, mentioned earlier, records can also be exported in RIS or TXT formats with the same batch limit of 100 000 records. The high export limit is an advantage for high volume searches, and the RIS format is advantageous for exporting and use with citation managers and systematic review screening software. A known issue with OpenAlex is the removal of non-open access abstracts from various publishers (specifically Springer and Elsevier) [12]. This has direct implications on both the search and the usability of the exported records for title-abstract screening. Therefore, it may be prudent to load the records from OpenAlex into screening software last, so that records from other sources are retained, in preference and based on import order, during deduplication.

**Fig. 5:**
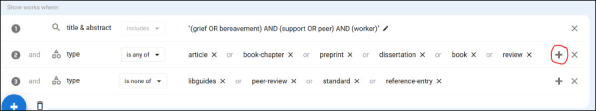
A search showing how to either include relevant Types or exclude irrelevant Types. Note the + symbol at the end of line 2 which allows a searcher to add more than one Type to the same line.

Due to the diverse types of information available in OpenAlex (e.g. peer review reports and LibGuides), evidence synthesis searchers will want to look at what “types” are being retrieved by their search. Searchers can either include the relevant types or exclude the irrelevant types–both are possible to implement (Figure 5).

One additional reason to use OpenAlex may be extensive content coverage, specifically the number of additional open access journals not covered by Web of Science core collection or Scopus [13].

## Strengths and weaknesses

Many of OpenAlex’s areas of strength come with corresponding weaknesses; these have been described in greater detail in the preceding sections, but are included as a summary below. The minimalist design of OpenAlex’s interface makes searching and navigation relatively easy, but this simplicity comes at the expense of advanced search features, which may render it unusable for some complex evidence synthesis searches.

Its broad scope and inclusivity make it one of the most comprehensive databases of scholarly (and other) works available, but can also lead to issues with messy and inconsistent metadata. The inclusion of information types that aren’t appropriate for evidence synthesis will need to be removed before exporting results.

Ultimately, OpenAlex’s openness, itself, is its biggest strength. Its integration with other open data sources (e.g. CrossRef, DOAJ, ORCiD) is a strong example of the power of open linked data, and presents an increasingly valuable alternative (or supplement) to the subscription-based resources libraries currently utilize.

OpenAlex has also fostered an active user support community where users can share experiences, troubleshoot issues, and work through new ideas [14]. As this community grows, it will hopefully help OpenAlex continue to improve and better serve its users.

## Cost

Access to OpenAlex via both the web application and API is currently available at no cost to users. OpenAlex has a freemium model for additional services such as more frequent data updates, API calls above 10 per second or 100 000 per day, priority support, and additional feature requests, training, or consultancy [15].

## Conclusion

Overall, OpenAlex is a promising option for both evidence synthesis searches and bibliometric analyses. A more extensive test and comparison against widely used subscription citation indexes and other openly accessible sources such as Lens.org and Google Scholar is needed.
